# On Cataract, Artificial Pupil, and Strabismus

**Published:** 1847-10

**Authors:** 


### Art. V.

*On Cataract, Artificial Pupil, and Strabismus.* By F. H. Brett, Esq., M.D., F.R.C.S.—*London,* 1847. 8vo, pp. 89. With four Plates.

The subjects discussed in this little work, are Cataract, Artificial Pupil, the Cure of Squinting by Operation, and the Phenomena displayed by Congenitally Blind Persons restored to Sight.

The author sets out by saying, that Celsus informs us, that among the physicians of Alexandria, there were many who practised the removal of cataract by operation, "especially one named Philoxene." Why Frenchify the name? Why not say Philoxenus? Perhaps Dr. Brett follows some general principle on this head, for we find he transmutes Mr. De la Garde, of Exeter, into Monsieur De la Garde; and even our worthy friend, Dr. John A. Robertson, of Edinburgh, appears as M. Robertson. After all, the whole of this Frenchification may be intended merely to prepare the reader for the coming announcement, that Dr. Brett has been in France, witnessing operations by M. Velpeau. But what is more important, we cannot agree with Dr. Brett's character of Philoxenus. He may have operated for cataract, but Celsus does not say so. Celsus merely states, that, being separated from the other parts of medicine, surgery began to have its peculiar professors, and received considerable improvements in Egypt, principally from Philoxenus, who treated of this part, (i. e. surgery), fully, and with great accuracy, in several volumes.

Besides having been in France, witnessing operations by M. Velpeau, Dr. Brett informs us, that he has been "in the vast peninsula of India; not only in Calcutta, but from thence to Lahore, Delhi, and other populous cities of the East." Need we wonder, when we are told, in true Oriental style, that he has "restored many thousands to sight?" And yet he joins M. Velpeau, in laughing at poor Lusardi, whose successful cases of couching amount to no more than 4168, out of 5034! What is this to "many thousands?"

We are next introduced by Dr. Brett to some "Anatomical Considerations;" the most remarkable of which is, that the figure of the crystalline "is that of a double convex lens, more convex anteriorly than posteriorly." We have always found it the reverse.

When objects appear multiplied to a cataractous eye, Dr. Brett thinks this may be attributed to opaque radii, passing from the centre to the circumference of the lens, which "act as the surfaces of a kaleidoscope;" —a similitude than which nothing could be more pat, provided we could only once understand how opaque radii are to act as surfaces.

Dr. Brett believes in the efficacy of medical treatment in cataract. This is rather ticklish ground to tread on, but as his remarks on the subject seem judicious, we think it right to let him speak for himself. We may just mention, as rather confirmatory of his views, that we have met with several cases, in which, on examining a suspected eye catoptrically, either no inverted image was discovered, or it was seen very obscurely; yet, on treatment being had recourse to, such as Dr. Brett recommends, vision has greatly improved, and the inverted image has appeared distinct. No doubt, the change might depend on the removal of some obscuration of the aqueous humour, or of the anterior surface of the anterior capsule, as well as of the lens itself, or of the posterior capsule ; for it is to be borne in mind, that, in estimating the changes that occur in the appearances of the images reflected from the eye in its several diseased states, it is necessary to take into account the state of the media through which we see them, as well as that of the surfaces which form the images. But let us hear Dr. Brett :—

"When the surgeon is consulted in the earlier stages of the disease, I am far from coinciding with the too prevalent opinion, that all medical treatment is of no avail. On the contrary, I believe that, under favorable circumstances, we may often succeed, if not in removing, at all events in greatly retarding, or even arresting, the further progress of the affection. If cataract is a disease of the constitution, if it is often hereditary, it is surely a rational practice to attempt to retard the progress of the disease by constitutional treatment. In the first class, whether of a palpably or obscurely inflammatory character, I believe that derivatives, the local abstraction of blood, counter-irritation, and a mild alterative course, combined with assiduous attention to the general health and constitution of the patient, is decidedly efficacious. On the other hand, whatever imparts tone to the system, improves the general health, and increases the capillary circulation, (not omitting the warm bath, with mild alteratives and tonic aperients,) is calculated to retard the advancement of the second or senile class of cataracts. Where there is a gouty or rheumatic diathesis, or a tendency to the predominance of lithic or phosphoric depositions in the urine, such remedies and regimen as tend to correct these states will be indicated. In debilitated habits, quinine, and other tonics, nutritious diet, with wine, regular exercise, and early hours, and the shower-bath." (p. 13.)

Dr. Brett recommends extraction to be performed with an instrument of the general figure of Beer's knife, but "increasing *suddenly* from the point to the shoulder, so that the section of the cornea may be completed by a single thrust before the point of the knife reaches the nose." He accordingly represents the edge and the back of the knife forming an angle of not less than 28°. This being a topic which we formerly discussed pretty fully (No. for April, 1844, p. 337, et seq.), we shall merely observe, that as the quantity of resistance to the penetration and progress of a knife through the cornea increases with the augmented angle of inclination of its edges, with one formed, as Dr. Brett recommends, it will be always difficult, and often impossible, to complete the section of the cornea. The resistance will often make it stick fast and immovable, before the section is half executed.

In perusing Dr. Brett's sections on cataract, from p. 15 to p. 26, we were struck with the suspicion, that surely we had met with the same matter, and in the same words before. On turning over some of the general treatises on the diseases of the eye, we found Dr. Brett had made a very unwarrantable use of Dr. Mackenzie's work—as the following parallel passages will show :

| Mackenzie, 3d Edition, p. 663. | Brett, p. 15. |
|---|---|
| "Double extraction decidedly exposes the eye to greater risk of inflammation. If we operate only on one eye, and allow it to recover, we may possibly observe, in the course of the operation and recovery, some particulars which will be essentially useful to us in conducting the second operation, or will even lead us to select a different and more suitable mode of operation for the second eye." | "Double extraction exposes the eye to greater risk. But by operating only on one eye at a time, we often observe, in the course of the operation and recovery, some particulars which will be essentially useful to us in conducting the second operation, or will even lead to a more suitable method of operating on the second occasion." |

Ib. p. 664.

"In cases of congenital cataract, ought the operation to be delayed till the patient has attained an age sufficient to enable him to understand the importance of an attempt to restore sight, or ought it to be practised during infancy? The answer decidedly is, operate in infancy."

Ib. p. 16.

"In cases of congenital cataract, ought the operation to be delayed until the patient has attained an age sufficient to enable him to give his assent, or ought it to be practised during infancy? It is greatly preferable to operate in infancy."

Ib. p. 681.

"The operation of extraction divides itself into three periods. In the *first*, the cornea is opened with the knife. In the *second*, the anterior hemisphere of the capsule is opened, or destroyed. In the *third*, the exit of the cataract, or the extraction properly so called, is accomplished."

Ib. p. 20.

"The operation may be conveniently divided into three periods. In the *first*, the incision of the cornea is effected. In the *second*, the anterior hemisphere of the capsule is opened, or rather destroyed as much as possible. In the *third*, the exit of the cataract, or the extraction, properly so called, is accomplished."

Ib. p. 684.

"In traversing thus the anterior chamber, let the operator bend his eye steadily on the point of *counter-punctuation*; if he do so, the point of the knife will be sure to follow, whereas, if he allows himself to be diverted to anything else, for instance, to what the edge of the knife is doing, he may miss his aim, and bring out the instrument at a wrong place."

Ib. p. 21.

"In traversing the anterior chamber, the operator should be very particular in directing his attention solely to the point of *counter-punctuation*; by so doing, the point of the knife is sure to follow; whereas, if he allows himself to be diverted by any object, he will very probably miss his aim."

It was not till we had noted four other plagiarisms fully as glaring as the above, viz. the passages commencing with "On pushing the knife, &c."—"If the aqueous humour has been, &c."—"In cases of capsulo-lenticular cataract, &c."—and "The posterior capsule of the lens, &c."—in pages 21 and 25, and corresponding with passages in pages 685-7-8 of Mackenzie, that coming to page 26, where Brett proceeds to consider the accidents during or after extraction, our eye caught the following announcement: "Here the rules recommended by Dr. Mackenzie correspond so much with my own, that with but few alterations or omissions, I can scarcely do otherwise than transcribe them." The next four pages and a half, then, are taken, with some slight verbal changes, from Dr. Mackenzie, till our author comes to inflammation as a consequence to be dreaded after extraction, when we find him disapproving, in acute cases, of mercury, lest it should check the adhesion of the cornea, and in sub-acute cases, recommending nutritious diet, alcoholic stimuli, carbonate of ammonia, and opium—following on this subject the distinctions and treatment of Mr. Tyrrell. It is sufficient to show the difficulty of distinguishing, by the symptoms, Mr. Tyrrell's acute from his subacute inflammation, that this gentleman recommends the giving of a dose of carbonate of ammonia, with or without opium, as may be thought proper, in order that the case may be pronounced sub-acute, if the patient experiences relief; acute, if the symptoms are aggravated; and the future steps of the treatment directed accordingly. (See Tyrrell's Work, vol. ii, p. 428.) The truth is, that old debilitated dram-drinkers seldom do well after any operation for cataract, and ought in general not to be meddled with. They are the subjects who suffer chiefly from inflammation after extraction; sometimes with the symptoms of Mr. Tyrrell's acute variety of the disease, sometimes with those of his sub-acute.

In making the section of the cornea, Dr. Brett advises the knife to be entered about a line from its margin, or midway between the margin of the cornea and the edge of the pupil. A line is too far from the sclerotica, so that the section will generally be too small, which is one of the most fruitful causes of inflammation.

For reclination or depression, Dr. Brett uses Scarpa's needle. Having passed it through the sclerotica, and carried it into the anterior chamber, he turns the point of the instrument backwards, and endeavours to break up the anterior capsule "near the ciliary processes, by carrying the point of the needle first in a circular direction around the periphery of the lens, and then by a crucial incision of the centre,"—a manœuvre which we believe to be impossible. The needle is held so fast by the sclerotica, that its point cannot be made to travel round the circumference of the lens. If it could, and the capsule was thus divided, no crucial incision could afterwards be made across its centre.

Dr. Brett must consider the retina as lining the whole extent of the choroid, for he tells us (p. 36), that to injure the retina is inevitable, at whatever distance we penetrate the sclerotic. Our opinion is, that the true or sentient retina ceases at the ora serrata. That some of the elements of the membrane, called retina, pass on to the ciliary processes, or even to the edge of the iris, we do not mean to deny; but we consider the wounding of the retina behind the ora serrata as likely to be injurious to vision, while puncturing it anteriorly to the ora serrata, or in other words, within $2\frac{1}{2}$ lines of the cornea, as of little or no consequence. Huschke will have it that the retina advances over the ciliary processes as far as the iris, but that there, at least in the fœtus, it does not form a free edge, but is reflected within itself, to form a second layer like a double nightcap.

In order to avoid the long ciliary artery, Dr. Brett directs us to enter the needle about a line below the transverse diameter of the eye; but as about a quarter of an inch from the iris, the long ciliary artery of the temporal side divides at an acute angle into two branches, an upper and a lower, it must be obvious that the surest way to avoid either of the branches is to enter the needle in the equator of the eye.

After describing the usual operation of division of the cataract through the cornea, our author goes on to state, that in fluid, soft, and capsular cataracts, he uses a sharp flat needle, and purposely allows the aqueous humour to escape, while he is performing a drilling movement of the needle. He says this is Mr. Tyrrell's "operation of drilling." Now Mr. Tyrrell's operation differed from this, not merely in preventing the escape of aqueous humour, as Dr. Brett mentions, but in being limited to cases of contracted pupil, adhering to an opaque capsule. For ordinary cases of soft cataract, we consider division through the sclerotica as the operation to be preferred, but were we induced to operate through the cornea, most assuredly we would follow the rule generally laid down, and particularly insisted on by Dr. Jacob, to retain, if possible, the whole of the aqueous humour. From an escape of this fluid, in division through the cornea, and from the iris being consequently pressed forwards into contact with the cornea, we have seen severe iritis ensue, ending in closure of the pupil.

At page 53, Dr. Brett says, "Although we must admit the truth of the general doctrine of the power of adjustment depending chiefly on the shifting of the lens, and although I acknowledge its doubly-refracting structure, and that its peculiar texture and gradually increasing density as it approaches its centre enables it to correct the spherical aberration of light, yet I must think that the loss of this body is not attended with so great a degree of deficiency with respect to these powers of the organ generally, *provided* the remaining parts of the eye, and especially the transparent media, are in the highest possible state of health." This last statement we may admit to a certain extent. No one has been known who could read an ordinarily printed book without the aid of a convex glass, after the removal of the crystalline; while such instances as that of the postilion, related by Sir William Adams, confirms the belief, that an eye, if young and otherwise uninjured, may accommodate itself in a very considerable degree to the loss of the crystalline, so far as distant objects are concerned; but pray, what has the doubly-refracting structure of the lens to do in the matter? Just as much as the double refraction of Iceland spar or the height of Strasburg steeple.

There seems to be a fatality attending every attempt to eulogise Cheselden.

"If astronomy," says Morand, "assures a kind of immortality to him who by chance becomes the discoverer of a single star, the same honour may well be bestowed upon a surgeon who knew by his genius to discover the whole heaven to a man born blind." In his haste to do justice to the great English surgeon, Morand has fallen into an error: for it was not in a case of congenital closure of the pupil, as Morand here supposes, that Cheselden first performed the operation for artificial pupil, but in a case of closed pupil after an operation for cataract.

Little less awkward is Mr. Guthrie. For though Cheselden published in the 'Philosophical Transactions' for 1728, two cases, in which he had formed an artificial pupil by incision of the iris, the one above and the other below the place of the natural pupil, which circumstances, with several others, are minutely related by him, Mr. Guthrie, in his work on 'Artificial Pupil,' published in 1819, after announcing that "the idea of forming an artificial pupil owes its origin to Mr. Cheselden, previous to whose time a closed pupil was considered irremediable," an assertion not quite correct,—gravely tells us that Cheselden "does not seem to have performed the operation on the person whose history he relates," a remark egregiously absurd.

"The illustrious Cheselden," says Dr. Brett, "had the distinguished merit of showing, in the last century, that sight might be restored in cases till then considered beyond the reach of art, by a new opening formed in a closed pupil. Various improvements and modifications have been introduced by modern surgeons since that period; and by the skill and ingenuity of a host of surgeons of the German, the Swiss, the French, and the English schools, following up the one idea of that one master-mind, we are now enabled to enrol the various operations for artificial pupil amongst the most brilliant in ophthalmic surgery."

Now, in the first place, the forming of "a new opening in a closed pupil" was no part of Cheselden's plan. This had been previously tried by Woolhouse, but without success. Cheselden's operation was a new opening in the iris, above or below the closed pupil. In the next place, to say that artificial pupil was "the one idea of that one master-mind," is a senseless oratorical flourish; for with no wish to set aside the old proverb, *Non unus omnia videt,* we may venture to affirm, that Cheselden had very many wise ideas in his head besides this, while what we have just mentioned about Woolhouse, shows that Cheselden was preceded by that oculist in attempts to restore sight in cases of closure of the pupil.

The first cases demanding the formation of an artificial pupil, noticed by Dr. Brett, he styles "occlusion of the pupil by an opacity of the cornea." This is a misnomer, as in such cases the pupil is not occluded, but merely covered. In the whole of the chapter on artificial pupil, there is an exceeding degree of negligence in the application of technical terms, and even in their spelling. Thus iridectomy, which means the formation of an artificial pupil by excision of a portion of the iris, is used as synonimous with incision; and we have coretomy and ceretomy, in place of corotomy. These are barbarous terms, but it would better become Dr. Brett to spell them correctly, than talk magniloquently of the "iridian diaphragm!"

To return to the first case requiring an artificial pupil, Dr. Brett states, that a central leucoma "is frequently complicated with a central cataract," adding that "this double obscurity of the dioptric media is explained in the following manner:—When the cornea becomes the seat of a central perforating ulceration, the aqueous humour escapes, the iris falls forwards, and the lens, in obedience to the contraction of the muscles, advances towards the cornea. The centre of the anterior part of the crystalline capsule comes in contact with, and receives the morbid products of the ulcer, inflames, and becomes opaque. When the ulcer closes, the aqueous humour again fills the two chambers, and the lens resumes its primitive position; but the corneal ulcer has been replaced by a dense white opacity, and a circumscribed opacity likewise occupies the capsule and lens itself." (p. 56.) Now, this formation of central cataract is, we believe, altogether a piece of imagination. We have never seen a case of the sort, and even Dr. Brett does not say he has ever seen one. Central cataract is not unfrequently met with in infants recovering from ophthalmia neonatorum, in whom there has been ulceration of the cornea, but without perforation; and therefore is not owing, at least in such cases, to the centre of the capsule coming into contact with the ulcer. We have also repeatedly seen the cornea perforated by ulceration, both in such infants and in other cases, but by the assiduous application of the extract of belladonna, the ulcer was brought to heal, the pupil expanded, no adhesions were left, and no central cataract.

With most authors who have written on the subject, Dr. Brett lays it down as a rule, that previous to undertaking an operation for artificial pupil, we should assure ourselves that the eye is able to distinguish between day and night. "Whatever," says he, "may be the density of the obstacle, the individual must be able to recognise so much light as to remove all doubt as to the possible complication of amaurosis." He adds, however, that "this principle admits of some exceptions," and concludes with the following observations, with which, with one small exception, we readily agree:—"In a patient, for example, labouring under a leucoma, the pupil being free behind the corneal obscurity, the operation would, doubtless, be contra-indicated, where the power of vision had become completely abolished. But if, under the same circumstances, a false membrane also obstructed the pupil, who can say that a soft cataract may not exist behind it, sufficient, of itself, to intercept every ray of light? An operation is, therefore, allowable in these doubtful cases." We grant that cases may occur, in which it may be proper to try the effect of forming an artificial pupil, although the patient does not perceive light. They must, however, be very rare, and we entirely dissent from the statement, that the presence of a soft cataract could, of itself, suffice to render the eye insensible to light. It must be a combination of partial opacity of the cornea, closure of the pupil, adhesions of the iris to an opaque capsule, and cataract, and such a combination only, which could produce insensibility to light, the retina remaining sound.

We are sorry, that we again meet, in this chapter, with whole sentences taken without acknowledgment from Dr. Mackenzie's Practical Treatise. Compare Brett, (pp. 60, 61, 64,) with Mackenzie, (pp. 738, 744.)

In those cases, in which an artificial pupil is demanded by a dense opacity of the cornea, with adhesion of the pupillary margin to the cicatrice, Dr. Brett recommends the cornea to be punctured with a broad needle, as the first step in the operation of excision. This we consider too narrow an instrument to make a sufficient incision. Mr. Tyrrell's hook is then to be passed through the puncture, "as far as the aperture of the pupil," and "introduced through the pupillary space." But how is this to be done, if the pupillary margin of the iris is adherent to the cornea? Under such circumstances, an incision, one fifth of an inch in length, should be made close to the margin of the cornea, the iris should then be punctured close to where it adheres to the cornea, and enough of the iris should be dragged out through the wound of the cornea by the hook, or a small pair of hooked forceps, to be cut off with the scissors.

Dr. Brett speaks very unfavorably of the operation of separation. The only case in which he admits this plan of operating is, when the cornea is the seat of a dense leucoma, with a small segment of iris uncovered. Here he advises a puncture to be made in the opaque part of the cornea, and if this is required towards the inner canthus, he does it with a curved lance-shaped knife, as a straight instrument would be interfered with by the nose. Yet he represents (Pl. I, fig. 1), the straight cataract knife dividing the cornea towards the inner canthus, as if this were quite a customary thing, whereas it must be very difficult, from the prominence of the internal angular process of the frontal bone. He then introduces through the puncture a small sharp hook, draws out a fragment of iris, and knips it off. He calls this "the only allowable modification of iridodialysis;" but it is not at all clear that it is an iridodialysis at all, he does not say that with the small sharp hook he separates any part of the iris from its adhesion to the choroid, and we rather think the operation is just a variety of excision. No greater injury will be done to the eye in this variety of excision than in any other. Perhaps, however, Dr. Brett, in explaining the want of success which is apt to attend this operation, refers not to the injury done by the operation, but to that inflicted on the retina by the original disease which has left the eye in so unfavorable a condition. On this point, he does not express himself very perspicuously.

The figures which accompany Dr. Brett's work are none of the best, but fig. 21 it is impossible to make anything of by itself, or to recognise, as we learn from the description (p. 73), that it is intended to represent a case where the cornea is transparent, and the iris, in consequence of iritis, adherent to the anterior capsule. For such a case, Dr. Brett recommends Mr. Tyrrell's operation of drilling, only he allows the aqueous humour to escape through the puncture made in the cornea. He adds to his account of the operation, that the pupil is preserved in a state of dilatation by the belladonna ointment; but as the pupil is adherent to the capsule, the belladonna will be of little use.

The chapter on Strabismus is very imperfect, occupying no more than five pages. The author tells us, that frequent observation convinces him that the operation "is not always successful," (p. 78); but in the course of the next six lines, he adds, that according to his "experience, the operation may be performed with almost invariable success." The latter announcement may lead one to expect some new and valuable hints as to the method of cutting for squinting, but nothing of the kind occurs. Dr. Brett seizes the conjunctiva, the subconjunctival fascia, and the tendon of the muscle, with two pairs of hooked forceps, one placed near the cornea, and the other near the caruncle, raises by this means a transverse fold, and snips it through with scissors, one blade being made to penetrate the fold and pass along the sclerotica, while the other is outside the fold. We consider this manipulation to be about as ugly a one as any which the squint-clippers have invented (and these have not been few), and as much more dangerous than some of them. Instead of the scissors "grating along the hard fibrous sclerotic coat," to use the elegant phrase of Dr. Brett, we can conceive the point, which must necessarily be sharp, to go suddenly through the sclerotic and choroid, so as to evacuate the vitreous humour, and we believe that this has happened more than once, in attempts to perform the section with scissors, or by a subconjunctival incision with a narrow knife. We doubt very much, whether the forceps will often raise up the muscle from the subjacent sclerotic; even when they raise the middle of it, the upper and lower fibres will be very apt to escape. There is not the smallest advantage gained by cutting the conjunctiva, fascia, and muscle, at one stroke. On the contrary, we think it not merely less violent, as well as safer, but greatly more conducive to success, to divide these textures separately.

Dr. Brett repudiates the hook for drawing the eye out of the inner canthus. It is seldom required. He separates the eyelids by an elastic wire speculum, which at once raises the upper and depresses the lower. The fingers of the assistant we deem preferable.

He cuts away the tendinous end of the muscle, which is left adhering near the cornea, after the section is made. We see no harm in this; but no part of the conjunctiva should be removed, as this leaves a broader cicatrice, permits fungous granulations to rise more readily from the bottom of the wound, and favours protrusion of the eye. Some operators are so solicitous about the conjunctiva healing closely, that they bring its edges together with a fine suture.

Dr. Brett's work is wound up with an appendix, "Concerning certain interesting Phenomena, manifested in individuals born blind, and in those having little or no recollection of that sense on their being restored to sight at various periods of life."

Metaphysicians have been led to expect, that a great degree of light might be thrown upon the peculiar provinces of the senses, and especially upon what belongs to sight and what to touch, by examining patients immediately after being operated on successfully for cataract. It seems quite certain, that a man born blind, being made to see, would at first have no idea of distance, nor magnitude, by sight. The sun and stars, the remotest objects as well as the nearer, would all seem to him to be in his eye, or rather in his mind. He would judge his thumb, with which he could hide a tower, or hinder its being seen, to be equal to that tower. Though his previous conceptions of tangible extension, divisibility, figure, and mobility might be considerable, he could have no idea of colour, nor of visible figure, nor of visible motion. To be of any use, however, to the metaphysician as a subject of experiment, for determining the truth or falsehood of the Berkeleian theory of vision, *First,* the man must have been totally blind; and, *secondly,* the acquirement of sight being accomplished, the experiments must be made the very first time his eyes are exposed to the light. Upon these points, metaphysicians have been grossly deceived, not being aware that cataract never deprives a person entirely of sight, and that in the course of recovery from the operations for cataract some amount of new visual experience is always obtained. The most completely developed cataract never prevents the patient from being able to tell, whether a candle is a few inches only from him, or several feet, or several yards; whether it is at rest, or in motion; whether it is moved vertically or horizontally. The generality of cataractous patients can distinguish the size, and shape, and colour, of most single objects, of about the size of the hand, when presented to them so as to be well contrasted with neighbouring objects, and their eyes turned from the light. Congenitally cataractous subjects are still more unfit than any others for the philosophical experiments in question—for this reason, that in them, the diseased lens does not grow in proportion to the rest of the eye, it remains small, or even shrinks, so as to become what oculists call *siliquose,* the consequence of which is, that the patients see very considerably, sometimes even to read large letters, by means of the rays of light admitted to the retina around the edge of the cataract.

Cheselden's expression that the blind lad upon whom he operated, and whose case has so often formed the basis of discussion, "was so far from making any judgment of distances, that he thought all objects touched his eyes," has also done much to mislead philosophers upon this head—an expression which Cheselden never could intend to be taken literally, and which, in fact, has no meaning, as no object of sight ever acts upon the eye in any way resembling contact. Even in the cases best calculated for testing the Berkeleian theory; those, namely, in which the cataracts have been large and dense, and the success of the operation sudden and perfect, the astonishment and agitation likely to be produced in the patient must be very apt to render him unable to give anything like an intelligent or satisfactory account of his new sensations.

None of the cases related by Dr. Brett are of any value in a philosophical point of view. It is amusing to find him remarking in regard to one of them, that "as his new sense could scarcely be said to have been fairly exercised more than fourteen days, further observations could not be made as to his judgment of distances, positions, forms, and motions!" Verily, the doctor has studied the subject pretty much in vain, if this is all he knows about it.

---

